# Alprazolam Reduces Inflammatory Cytokine Production in Pancreatic Cancer–Associated Fibroblasts

**DOI:** 10.1158/2767-9764.CRC-25-0472

**Published:** 2026-05-06

**Authors:** Hunter D. Reavis, Aditi H. Chaubey, Amanda L. Smythers, Arwen A. Tisdale, Amanda Tracz, Alphonse N. Dimeck, Kathryn E. Maraszek, Eduardo Cortes Gomez, Joao A. Paulo, Subhamoy Dasgupta, Steven P. Gygi, Michael E. Feigin

**Affiliations:** 1 Department of Pharmacology and Therapeutics, Roswell Park Comprehensive Cancer Center, Buffalo, New York.; 2 Department of Cancer Biology, Dana-Farber Cancer Institute, Boston, Massachusetts.; 3 Department of Cell Biology, Harvard Medical School, Boston, Massachusetts.; 4 Comparative Oncology Shared Resource, Roswell Park Comprehensive Cancer Center, Buffalo, New York.; 5 Department of Cell Stress Biology, Roswell Park Comprehensive Cancer Center, Buffalo, New York.; 6 Department of Biostatistics and Bioinformatics, Roswell Park Comprehensive Cancer Center, Buffalo, New York.; 7 Department of Biostatistics, State University of New York at Buffalo, Buffalo, New York.

## Abstract

Pancreatic ductal adenocarcinoma (PDAC) diagnoses are often accompanied by a number of physical and psychologic symptoms, including anxiety and depression. As a result, many patients are prescribed anxiolytics such as benzodiazepines (BZD) that have unintended effects on the tumor. Previous work from our lab has highlighted that structural differences between BZD compounds may be responsible for different clinical outcomes influenced by their effects on cancer-associated fibroblasts (CAF) within the PDAC tumor microenvironment (TME). In this study, we demonstrate that the commonly prescribed N-substituted triazolobenzodiazepine alprazolam (ALP) abrogates the production of proinflammatory cytokines, including CCL2, CXCL12, interleukin 6 (IL6), and IL8, in human CAFs. This phenotype is unique only to azole-containing BZDs, including midazolam. The ability of ALP to regulate proinflammatory cytokine production is maintained *in vivo* as ALP-treated mice bearing pancreatic tumors exhibited reductions in IL6 within the tumor interstitial fluid. Mechanistically, an unbiased phosphoproteomic approach revealed that ALP abrogates Toll-like receptor 4–mediated cytokine production in CAFs. These findings cumulatively support that ALP dampens CAF-mediated inflammatory signaling within the PDAC TME.

**Significance:**

The data in the present study focus on the characterization of the effects of ALP on CAF output and provide a compelling basis to suggest that ALP may have a broad impact on remodeling the pancreatic cancer immune landscape and therapeutic response.

## Introduction

Pancreatic cancer is one of the most aggressive solid tumor malignancies, with an overall 5-year survival rate of 13% (1). At early stages, this disease presents with nonspecific symptomatology, and as a result, more than 48% of patients with pancreatic cancer are diagnosed with advanced disease, at which point the 5-year survival rate is as low as 3.2% (2). The most common subtype of pancreatic cancer is pancreatic ductal adenocarcinoma (PDAC), comprising up to 90% of cases, with the poorest prognosis of all pancreatic neoplasms (3–5).

The dismal prognosis of this disease, as well as its impact on quality of life, gives rise to a high incidence of anxiety and/or depression among patients with pancreatic cancer (∼30%; refs. 6–9). In approximately 50% of cases, these symptoms are managed with pharmacotherapy such as selective serotonin reuptake inhibitors (SSRI) and benzodiazepines (BZD; ref. 9). The choice between SSRIs and BZDs is often subjective, heavily depending on the discretion of the prescribing physician. Generally, SSRIs are preferred for the treatment of general anxiety and depression, whereas BZDs are considered for anticipatory nausea relief. Over the past 25 years, it has become well appreciated that some of these compounds, particularly SSRIs, may have unintended effects on the tumor and tumor microenvironment (TME; refs. 10–16) although investigations in the context of PDAC remain sparse. Therefore, it is important to better understand how these commonly prescribed drugs affect intrinsic PDAC tumor biology and therapeutic response.

A recent study from our lab reported that more than 40% of patients with PDAC at Roswell Park Comprehensive Cancer Center are prescribed BZDs (17). BZDs are a collective group of compounds that modulate the activity of γ-aminobutyric acid A (GABA-A) receptors in the central nervous system and consequently exhibit clinical utility as sedatives, anxiolytics, and anticonvulsants (18). Although these compounds share a basic structure (benzene ring + diazepine ring), they are structurally distinct and can be further classified by the presence or absence of different modifications to the nitrogen atoms of the BZD core scaffold (N-substituted vs. N-unsubstituted). We have previously reported that these subtle structural differences may be associated with different outcomes in PDAC (17).

More specifically, our epidemiologic analyses revealed that patients with PDAC who were prescribed the N-unsubstituted BZD lorazepam (LOR) exhibited poorer progression-free survival when compared with patients who did not receive any BZD prescription (17). Conversely, patients with PDAC who were prescribed the N-substituted BZD alprazolam (ALP) exhibited improved progression-free survival. Functional follow-up studies revealed that one possible explanation for these findings could be the differential regulation of inflammatory signaling via interleukin 6 (IL6), an inflammatory cytokine primarily secreted by cancer-associated fibroblasts (CAF) with well-defined roles in the development and progression of PDAC (19–24). Although LOR treatment led to an elevation of IL6 secretion in CAFs, ALP treatment reduced IL6 secretion below baseline, suggesting that ALP may exhibit anti-inflammatory effects *in vitro*.

In the present study, we set out to characterize the effects of ALP on the inflammatory secretome of CAFs, a key component of the TME that regulates fibrosis and immune exclusion in PDAC (25).

Herein, we report that in addition to IL6, ALP treatment also reduces the production of important immunomodulatory cytokines such as CCL2, CXCL12, and IL8 in CAF cells. Importantly, this reduction in cytokine production seems to be restricted to BZD compounds containing an azole group (ALP, midazolam), further emphasizing the structure-specific effects of BZDs. We also take an *in vivo* approach using an orthotopic PDAC mouse model to find that ALP-treated tumors exhibited lower levels of IL6 in the tumor interstitial fluid (TIF), indicative of an ALP-dependent change in the intratumoral cytokine milieu. Mechanistically, we demonstrate that modulation of the known off-target receptors platelet-activating factor receptor (PTAFR) and translocator protein (TSPO) can only partially phenocopy ALP treatment. We also report a broader role for ALP in regulating Toll-like receptor 4 (TLR4) signaling, whereby ALP treatment decreases transcript levels of inflammatory cytokines exacerbated by TLR4 agonism. Altogether, these results support the notion that ALP exerts anti-inflammatory effects on the PDAC TME.

## Materials and Methods

### Reagents and materials

All compounds were purchased from Cayman Chemical, Sigma-Aldrich, or Thermo Fisher Scientific and diluted in sterile dimethyl sulfoxide (Corning, 25950CQC) or sterile UltraPure water (Invitrogen, 10977023) where applicable. Detailed information for each compound can be found in Supplementary Table S1.

### Cell culture

C7-TA-PSC immortalized human (male) CAFs were a gift from Dr. Edna Cukierman (Fox Chase Cancer Center, Philadelphia, PA). 4175 murine CAFs were established in the lab of Dr. Jason Pitarresi (University of Massachusetts, Worcester, MA) by flow cytometric selection of YFP cells from a Kras^LSL-G12D/+^; Trp53^LSL-R172H/+^; YFP^LSL^; Pdx1-Cre tumor in a male C57BL/6 mouse over two passages. Fibroblast phenotypes were then further confirmed via flow cytometry staining for α-smooth muscle actin or podoplanin. PANC1 human PDAC cells (male; RRID:CVCL_0480) were purchased from the American Type Culture Collection in March 2021. KPC1245 murine PDAC cells were derived from a Kras^LSL-G12D/+^; Trp53^LSL-R172H/+^; Pdx1-Cre tumor in a female C57BL/6 mouse in the lab of Dr. David Tuveson (Cold Spring Harbor Laboratory, Cold Spring Harbor, NY). No further authentication was conducted on the aforementioned cell lines. All cell lines were cultured in 10 cm tissue culture–treated dishes (Fisher Scientific, 08-772-22) at 37°C and 5% CO_2_ in DMEM with glucose, L-glutamine, and sodium pyruvate (Corning, 10-013-CV) supplemented with 10% FBS (Corning, 35-011-CV) and 1% penicillin/streptomycin (Corning, 30-002-CI). Upkeep cultures were maintained at ∼80% confluence. All experiments were conducted with cells of less than 30 passages from receipt. Cell supernatants were routinely tested for mycoplasma and tested negative. The date of the last mycoplasma testing was April 3, 2025.

### Cytokine array

A total of 500,000 C7-TA-PSC cells were cultured in respective wells of a six-well plate (Thermo Fisher Scientific, 130184) for 24 hours before the media was refreshed with 2 mL of complete DMEM (10% FBS + 1% penicillin/streptomycin) containing either DMSO or 20 μmol/L ALP. Cells were incubated for an additional 24 hours, and conditioned media was collected via centrifugation at 1,000 rpm for 3 minutes to remove cells and large debris. One milliliter of conditioned media was utilized for sample preparation according to the manufacturer’s protocol (R&D Systems, ARY005B). Membranes for both treatment conditions were imaged simultaneously using a Bio-Rad ChemiDoc MP system for 5 minutes using the “Hi Sensitivity” application. Relative cytokine levels were quantified using ImageLab software (Bio-Rad). Briefly, each array dot was mapped out using a fixed volume round outline, including dots for the manufacturer’s blanks. After subtracting the average background signal for each membrane from the individual cytokine dot signal, values were divided by 10^4^ and reported as the total pixel intensity in arbitrary units.

### Analysis of cell viability

Cells were plated and treated as described above. After 24 hours of treatment with either DMSO or 20 μmol/L ALP, the entirety of the media (2 mL) containing nonadherent cells was collected from the respective wells and transferred to a sterile 15 mL conical (Corning, 430790). Adherent cells were then washed once in 1× DPBS (Corning, 20-031-CV), and 500 μL of 0.25% trypsin (Corning, 25-053-CI) was added to each well. After a 5-minute incubation at 37°C and 5% CO_2_, trypsin was neutralized with 1 mL of complete DMEM, and the cell suspension was transferred to the 15 mL conical containing the nonadherent cells for each of the respective conditions. Cells were pelleted at 1,000 rpm for 3 minutes, the supernatants were aspirated, and each pellet was resuspended in 500 μL of complete DMEM. Suspensions were transferred to a counting cup (Beckman Coulter, 383721) and loaded into a Vi-CELL XR Cell Viability Analyzer. Prior to uptake by the machine, suspensions were mixed using a 1,000 μL pipette to prevent cells from settling in the counting cup. Cells were stained with an internal trypan blue reagent (Beckman Coulter, B94987) for dead cell exclusion, and cell counts from 50 fields of view were averaged together for each biological replicate. Cell count per volume (cells/mL) values were divided by 2 to account for the concentrated 500 μL input.

### Microscopy

For phase-contrast images, cells were plated and treated as described above. Twenty-four hours after treatment with 20 μmol/L ALP or vehicle control, each respective well of the six-well plate was visualized, and representative images were taken using an Olympus IX73 microscope at 10×. Scale bars represent 200 μm.

For fluorescent phalloidin staining of F-actin filaments, 50,000 cells were plated into 2 wells of an eight-well μ-Slide with high walls for microscopy (ibidi, 80806) on day 0. On day 1, the media was changed and replaced with fresh media containing either 20 μmol/L ALP or DMSO for the respective wells. Twenty-four hours later, the media was removed, and the cells were fixed using 4% paraformaldehyde in PBS (Thermo Fisher Scientific, J61899.AK) for 15 minutes. The cells were washed 3 times in PBS prior to permeabilization with 0.1% Triton X-100 (Sigma-Aldrich, X100) diluted in PBS for 15 minutes. Cells were washed 3 times in PBS and incubated with Alexa Fluor 488 Phalloidin (Thermo Fisher Scientific, A12379) stain (diluted to 1× in PBS from 40× methanol stock) for 30 minutes in the dark per the manufacturer’s instructions. After three final PBS washes, cell nuclei were stained using two drops of Fluoroshield with DAPI (Sigma-Aldrich, F6057) per well. Slides were imaged using a tetramethylrhodamine filter to visualize the phalloidin stain (green) and DAPI to visualize nuclei (blue). Images were acquired using a KEYENCE BZ-X710 fluorescence microscope at 20×. Scale bars represent 50 μm.

### RT-qPCR

Adherent cells were washed once in 1× DPBS and diethyl pyrocarbonate water (Thermo Fisher Scientific, AM9916) prior to lysis and RNA isolation using the Norgen Total RNA Purification Plus Kit (48300). RNA concentration and quality were assessed via NanoDrop for samples with 260/280 > 2 and 260/230 > 1.75. One microgram of RNA was reverse transcribed using the iScript cDNA Synthesis Kit (Bio-Rad, 1708891). RT-qPCR was performed in biological and technical triplicate unless otherwise noted, using the iTaq Universal SYBR Green Supermix (Bio-Rad, 1725121). Ten microliters of each sample was analyzed on the CFX Opus 96 Real-Time PCR system (Bio-Rad) using default settings for PrimePCR primers. Primers were all purchased from Bio-Rad and are annotated in Supplementary Table S2. C_t_ values for each technical replicate were averaged and, unless otherwise noted, used for ΔΔC_t_ analysis to calculate the fold change in gene expression relative to GAPDH C_t_ values (ΔC_t_) and the C_t_ values for the DMSO controls (ΔΔC_t_). Fold change (relative RNA expression) was calculated as 2^−ΔΔCt^. This method was employed for each biological replicate individually such that the fold change for the vehicle control in all three biological replicates is equal to 1 unless otherwise noted.

### Enzyme-linked immunosorbent assays

#### Human CAF enzyme-linked immunosorbent assays

On day 0, 500,000 C7-TA-PSC cells were plated into respective wells of a 6-well plate. On day 1, fresh media was added to each well with 20 μmol/L of the indicated BZD or DMSO control. Twenty-four hours later, conditioned media was isolated as described above. The supernatant was then aliquoted and stored at −80°C before use in each assay (<2 weeks). For each enzyme-linked immunosorbent assay (ELISA), conditioned media was thawed on ice, and each biological replicate (*n* = 3) was tested in technical duplicate. CCL2 (Invitrogen, BMS281), CXCL12 (Invitrogen, EHCXCL12A), IL6 (Sigma-Aldrich, RAB0306), and IL8 (Invitrogen, KHC0081) ELISAs were carried out per the manufacturer’s protocols. Samples were diluted 1:10 for use in the CCL2 ELISA to avoid exceeding the maximum limit of detection. Standard curves were generated to calculate total protein levels, and data were normalized to the concentration of the DMSO control.

#### Murine CAF ELISAs

On day 0, 250,000 4175 cells were plated into respective wells of a six-well plate. On day 1, fresh media was added to each well with 20 μmol/L of ALP or DMSO control. Twenty-four hours later, conditioned media was isolated and stored as described above. For each ELISA, conditioned media was thawed on ice, and each biological replicate (*n* = 3) was tested in technical duplicate. CCL2 (Invitrogen, BMS6005) and IL6 (Invitrogen, BMS603-2) ELISAs were carried out per the manufacturer’s protocols. Samples were diluted 1:10 for use in the murine CCL2 ELISA. Standard curves were generated to calculate total protein levels, and data were normalized to the concentration of the DMSO control.

### PANC1 cell culture for cytokine RNA analysis

On day 0, 250,000 PANC1 human PDAC cells were plated into respective wells of a six-well plate. On day 1, media was aspirated and replaced with media containing DMSO or 20 μmol/L ALP. Cells were incubated for 24 hours prior to RNA isolation as described above.

### Orthotopic pancreatic allografts and *in vivo* drug treatment

All *in vivo* experiments were conducted in accordance with Institutional Animal Care and Use Committee (IACUC) guidelines (Protocol #1381M). On the day of surgery, 10-week-old female C57BL/6J mice (RRID:IMSR_JAX:000664) were anesthetized using isoflurane. Each mouse received a subcutaneous bolus of 500 μL of 0.9% saline (Sigma-Aldrich, S8776) and a long-acting analgesic (Ethiqa). The surgical site was shaved and disinfected using chlorhexidine, 70% EtOH, and betadine. Mice were transferred to a sterile surgical plane on a heating pad and draped prior to surgery. Small incisions were made in the skin and the peritoneal membrane in order to exteriorize the spleen and pancreas. A 20 μL suspension of serum- and antibiotic-free DMEM containing 10,000 KPC1245 cells was injected into the head of the pancreas using a Hamilton syringe (Hamilton, 80501). After interiorizing the spleen and pancreas, the peritoneal membrane was closed using 5-0 Vicryl sutures, and wound clips were applied to the skin. Mice were monitored for 72 hours for postoperative recovery before beginning daily i.p. injections of DMSO or ALP (0.5 mg/kg). For drug preparation, ALP was first diluted in DMSO to create a stock of 10 mg/mL. On the day of injection, the stock concentration was diluted to 50 μg/mL in 0.9% saline, and syringes were loaded with 200 μL of solution per 20 g of mouse body weight. Mice were weighed every 5 to 7 days to maintain proportional dosing.

### Collection and analysis of serum from whole blood

In accordance with IACUC standards, mice were euthanized using CO_2_ prior to intracardiac puncture. Whole blood was transferred to a microcentrifuge tube (Thermo Fisher Scientific, 1149X91) and allowed to coagulate at room temperature for 30 minutes prior to centrifugation at 10,000 rpm for 10 minutes. Supernatant (serum) from each sample was aliquoted into microcentrifuge tubes (25 μL/tube) and stored at −80°C (<2 weeks) prior to use in murine ELISAs (see “Murine CAF ELISAs”). Input volumes for serum ELISAs reflected 1 μL per 1 g of body weight to normalize for size differences between mice. Unlike conditioned media, serum was not diluted for use in the CCL2 ELISA.

### Collection and analysis of TIF

Primary pancreatic tumors were isolated from the peritoneal cavity and washed briefly with cold saline. Tumors were blotted with Kimwipes (Fisher Scientific, 06-666) to remove excess saline and were placed onto 20 μm Spectra/Mesh filters (Spectrum, 148134) secured on top of a 50 mL conical tube (Falcon, 352070). Lids were secured on each tube and transferred to a refrigerated centrifuge (4°C) for a 10-minute, 1,500 rpm spin. Flow-through (TIF) from each tumor (∼25 μL) was aliquoted into cryovials (VWR, 10018-738) and flash-frozen in liquid nitrogen. Samples were stored at −80°C (<2 weeks) prior to use in murine ELISAs (see “Murine CAF ELISAs”). Input volumes for TIF ELISAs reflected 1 μL per 0.1 g of tumor to normalize for tumor size. TIF was not diluted for use in the CCL2 ELISA.

### Receptor/ligand dose escalation assays

A total of 500,000 C7-TA-PSC cells were plated into each well of a six-well plate and incubated overnight. On the day of treatment, media was prepared in 15 mL conicals containing the vehicle or designated concentration of each compound (GABA, PK11195, WEB2086, ALP). Compound diluents were utilized as the vehicle for each respective compound. Media was removed from the plated cells and replaced with fresh media containing the indicated drug concentration. Cells were incubated for an additional 24 hours prior to collection for RNA isolation.

### Analysis of human PDAC single-cell sequencing dataset

Normalized single-cell RNA sequencing (scRNA-seq) data for human pancreatic adenocarcinoma were directly obtained from the Chijimatsu and colleagues study (26). The original authors performed all upstream processing steps, including raw read alignment, unique molecular identifier counting, initial quality control, and normalization. The preprocessed data, typically provided in a Seurat object format or similar, were loaded into an R statistical computing environment (R version 4.2.0). Downstream analyses of the single-cell data were conducted using the Seurat R package (version 5.0.0; ref. 27).

Upon loading the scRNA-seq data into the R session, normalized expression data for a predefined list of genes of interest were programmatically retrieved from the Seurat object. Comprehensive gene reports were generated to systematically examine the expression of these genes across various metadata factors. These factors included, but were not limited to, the specific project contributing the data, the disease type status (e.g., tumor vs. normal tissue), and cellular subtype (e.g., fibroblast vs. ductal), as classified in ref. (26). Gene reports typically comprised summary statistics (e.g., mean expression level, percentage of cells expressing the gene) and visualizations such as Uniform Manifold Approximation and Projection for Dimension Reduction plots, violin plots, and box plots or feature plots to illustrate gene activity and distribution within different cellular and sample contexts.

To visually highlight general differential expression patterns of the genes of interest, heatmaps were produced. These heatmaps depicted the normalized expression levels of the selected genes across individual cells or aggregated cell clusters, providing a comprehensive overview. Emphasis was placed on generating heatmaps that specifically compared gene expression profiles between tumor and normal samples, allowing for a broad assessment of disease-associated expression changes. Comparison heatmaps were created focusing on the fibroblast population based on the data’s annotation.

### siRNA transfection

On day 0, 500,000 cells were plated into two wells of a six-well plate. Twenty-four hours later, cells were transfected with either a nontargeting control siRNA pool (siNTC; Horizon, D-001206-13-05) or a SMARTpool of four siRNAs targeting TSPO (Horizon, M-009559-03-0005) using Lipofectamine RNAiMAX reagents (Invitrogen, 13778075). Briefly, 5 nmol of each lyophilized pool was reconstituted in 1× siRNA buffer (Horizon, B-002000-UB-100) to create a stock of 20 μmol/L siRNA that was stored at −20°C. On the day of transfection, 1.25 μL of the 20 μmol/L siRNA stock was diluted in 200 μL of Opti-MEM (Gibco, 31985062), along with 2 μL of RNAiMAX reagent. This mixture was then vortexed thoroughly for 30 seconds to ensure mixing, briefly centrifuged to pool all liquid, and incubated at room temperature for 15 minutes. The media on the cells was replaced with 800 μL of Opti-MEM at this time. The siRNA solution (∼203 μL) was then added to the respective well for each siRNA condition (NTC vs. TSPO). Four hours later, the cells were replenished with 1.5 mL of antibiotic-free culture media (DMEM + 10% FBS).

### ALP time courses

A total of 500,000 C7-TA-PSC cells were plated into each well of a six-well plate and incubated overnight. At time point 0, media was removed from the cells and replaced with fresh media containing 20 μmol/L ALP or DMSO equivalent. Cell pellets were collected via trypsinization at each time point and frozen at −80°C prior to RNA isolation.

### Experimental design graphics

All experimental design graphics were created using BioRender (RRID:SCR_018361, https://BioRender.com).

### Phospho-enrichment sample preparation

A total of 2,500,000 C7-TA-PSC cells were plated into 10 cm dishes for each respective condition and incubated overnight. One hour before treatment with ALP, media was refreshed on the cells to remove any constitutive signals that had accumulated after cell seeding while allowing the cells to equilibrate from the media change. One hour later, media was refreshed once again with 20 μmol/L ALP, and cell pellets were collected via trypsinization and frozen at each consecutive time point. For phospho enrichment, cell pellets were lysed with 8 mol/L urea dissolved in 150 μL lysis buffer [50 mmol/L 4-(2-hydroxyethyl)piperazine-1-propanesulfonic acid (EPPS; pH 8.5), 1% SDS, 1 PhosSTOP (Roche), 1 cOmplete Mini EDTA-free Protease Inhibitor Cocktail Tablet (Roche)] before clarifying with 10 minutes of centrifugation at 4°C and 16,000 × *g*. The supernatant was quantified using the CB-X protein assay (G-Biosciences). All samples were diluted to 1 mg/mL with water, reduced with 5 mmol/L DTT for 30 minutes, and alkylated with 15 mmol/L iodoacetamide in the dark for 30 minutes.

Samples were desalted using the SP3 method (28), with a 1:1 mixture of two magnetic beads (Cytiva, 45152105050250 and 65152105050250). After mixing a 1:10 mixture of beads and protein, the protein was precipitated through the addition of 2× volume 100% ethanol for 15 minutes, before washing with 80% ethanol twice. The beads were then incubated in 75 μL of 50 mmol/L EPPS buffer with 1 μg of MS Trypsin (Thermo Fisher Scientific) overnight at 37°C, shaking at 800 RPM. Peptides were labeled by adding anhydrous acetonitrile (Sigma) to 30% v/v, followed by TMTpro (Thermo Fisher Scientific, A44520) reagent. One percent of each labeled sample was combined and analyzed unfractionated to ensure labeling efficiency was >97%, and all channels were combined. The combined sample was desalted with a 100 mg C18 Sep-Pak (Waters) following the manufacturer’s instructions before drying with a vacuum centrifuge. Peptides were reconstituted and enriched for phosphopeptides using an Fe-NTA spin column (Thermo Fisher Scientific, A32992). The flow-through was enriched for further phosphopeptides using a TiO_2_ spin column (Thermo Fisher Scientific, A32993), both following the manufacturer’s instructions. Samples were desalted using C18 StageTips for LC/MS-MS analysis.

### Liquid chromatography and mass spectrometry data acquisition

Mass spectrometry data were collected using an Orbitrap Exploris 480 mass spectrometer (Thermo Fisher Scientific) coupled to an nLC-1200 liquid chromatograph. Peptides were separated on a 100 μm inner diameter microcapillary column packed with ∼35 cm of Accucore C18 resin (2.6 μm, 150 Å, Thermo Fisher Scientific). For each analysis, we loaded ∼2 μg onto the column. Peptides were separated using a 150-minute gradient of 5% to 29% acetonitrile in 0.125% formic acid with a flow rate of 325 nL/minute. The scan sequence began with an Orbitrap MS1 spectrum with the following parameters: resolution 60K, scan range 350 to 1,350, automatic gain control (AGC) target set to “standard,” maximum injection time set to “auto,” and centroid spectrum data type. We use a cycle time of 1 second for MS2 analysis, which consisted of high-energy collision dissociation (HCD) with the following parameters: resolution 45K, AGC 200%, maximum injection time 125 milliseconds, isolation window 0.6 Th, normalized collision energy 35%, and centroid spectrum data type. Dynamic exclusion was set to automatic. The sample was analyzed twice, once with an FAIMS compensation voltage (CV) set at −40, −60, and −80 V and a second injection with a CV set at −30, −50, and −70 V.

### Data analysis for mass spectrometry

In-house software tools were used for database searching and reporter ion quantitation, as previously reported (29). MS/MS spectra were assigned using the Sequest algorithm and matched to a UniProt human database (compiled October 2024), with both forward and reverse sequences combined to enable the target-decoy strategy (30, 31). Database searches were performed with static modifications of cysteine alkylation and tandem mass tag (TMT) on the peptide N-termini and lysine residues. Variable modifications included oxidation of methionine and phosphorylation of serine, threonine, and tyrosine. Neutral phosphate loss was searched for and identified as well. The precursor ion tolerance was 50 ppm, and the fragment ion tolerance was 0.8 Da (for HCD). Sequest matches were filtered using linear discriminant analysis, as previously reported, first to a dataset-level error of 1% at the peptide level based on matches to reversed sequences (31).

### Peptide quantification

TMT reporter ions were quantified as previously published (32). Briefly, TMT signals were isolated with a filter of 0.004 Da and corrected for isotope impurities according to the manufacturer’s instructions. Peptides were only considered quantifiable if the total signal-to-noise for all channels was >160.

### Statistical analyses of phosphopeptide site localization and quantitation

Phosphorylation sites were assigned to specific residues using a modified version of the AScore algorithm. Phosphorylation sites with AScore values >13 (*P* ≤ 0.05) were considered confidently localized to a particular residue (29). The Student *t* test was used to assign confidence to changes in phosphopeptide abundance.

### Kinase prediction analysis

Phosphopeptides were input into the PhosphoSitePlus Phosphoproteomic Enrichment Tool (version 0.1.0, Legacy Version; refs. 33, 34) as a tab-delimited text file with the annotated phosphoacceptor, log_2_ (fold change), and *P* values in respective columns for differential expression–based analysis. Cutoffs for log_2_ (fold change) and *P* value were set at 0.322 and 0.05, respectively. Enrichment analysis was run for canonical and noncanonical serine/threonine and tyrosine kinases (total = 396 kinases) using default settings.

### Gene Ontology enrichment

A list of proteins for all significantly differentially regulated phosphopeptides [|log_2_ (fold change)| ≥ 0.322, −log_10_ (*P* value) > 1.3] was uploaded to EnrichrKG (35) for each time point. The output for this analysis yielded the top five pathways from the Kyoto Encyclopedia of Genes and Genomes 2021 (RRID:SCR_012773), Gene Ontology (GO) Biological Process 2021 (RRID:SCR_002811), and Reactome 2022 datasets (RRID:SCR_003485). Graphics depicting *z*-scores and *P* values for the resulting 15 pathways were created using Origin 2025 software.

### Lipopolysaccharide agonism assay

A total of 500,000 C7-TA-PSC cells were plated into each well of a six-well plate and incubated overnight. At the time of treatment, the media was removed from the cells and replaced with fresh media containing either DMSO, 20 μmol/L ALP, and/or 2.5 μg/mL lipopolysaccharide (LPS; 1:1,000). The cells were incubated for an additional 24 hours prior to collection for RNA isolation.

### Statistical analyses

All experiments were conducted in technical and biological triplicate, and each data point on bar graphs represents one biological replicate unless otherwise noted. Error bars reflect mean ± SD. All statistical analyses were conducted in GraphPad Prism 10 (RRID:SCR_002798). For analyses comparing only two groups, an unpaired, two-tailed Student *t* test was utilized. Analyses of a single variable across more than two groups were conducted using one-way ANOVA with a Dunnett’s correction when experimental groups were compared only with the control and a Tukey correction when experimental groups were compared with all groups. *P* values of <0.05 were considered significant.

## Results

### ALP alters the inflammatory secretome of PDAC CAFs *in vitro*

**Figure 1.**
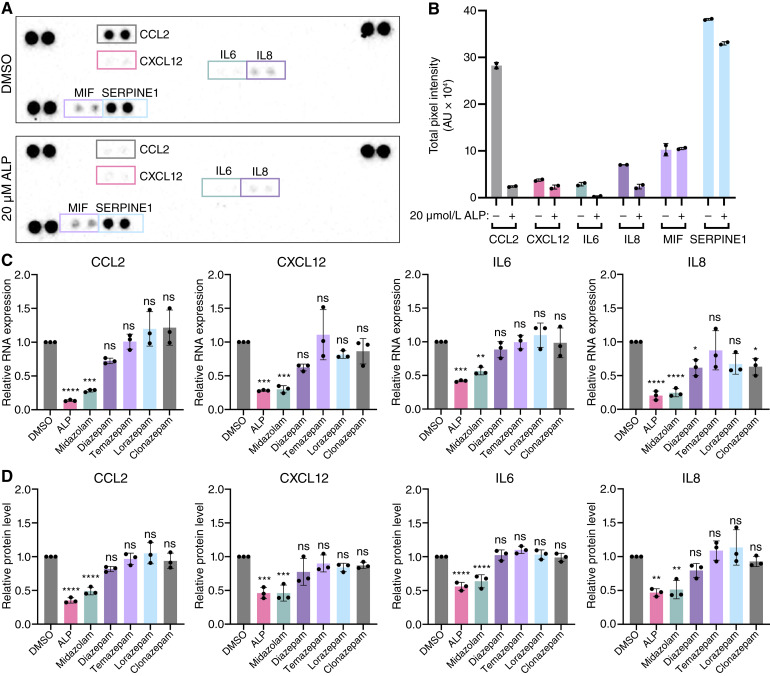
ALP alters the inflammatory secretome of PDAC CAFs *in vitro*. **A,** Annotated images of cytokine array membranes following incubation with conditioned media from C7-TA-PSC cells treated with 20 μmol/L ALP or DMSO for 24 hours. **B,** Quantification of the total pixel intensity for cytokines visualized in **A**. Data points reflect values of each technical duplicate from a single biological replicate (*n* = 1). AU, arbitrary units. **C,** RT-qPCR quantification of the fold change in cytokine RNA expression from C7-TA-PSC cells after treatment with 20 μmol/L of each BZD relative to the DMSO control. Data were collected in technical duplicate and biological triplicate (*n* = 3). **D,** Quantification of cytokine protein levels detected by individual ELISAs using the conditioned media from cells treated as in **C**. Data were collected in technical duplicate for three biological replicates (*n* = 3). Data in **B–D** are represented as mean ± SD. All statistical analyses in **C** and **D** were conducted using one-way ANOVA with Dunnett correction for multiple comparisons. *, *P* < 0.05; **, *P* < 0.01; ***, *P* < 0.001; ****, *P* < 0.0001.

Immortalized human CAFs (C7-TA-PSC) were treated with DMSO or 20 μmol/L ALP for 24 hours, and conditioned media was collected to assess the relative abundance of 36 different cytokines in a membrane-based immunoassay. In addition to IL6, ALP treatment led to a reduction in protein levels of CCL2, CXCL12, IL8, and SERPINE1 within the conditioned media (Fig. 1A and B).

In order to assess whether the overall reduction in cytokine production was attributable to changes in cell survival following ALP treatment, we evaluated the viability and morphology of the cultures. We observed no significant decrease in overall cell count or viability between treatment groups (Supplementary Fig. S1A and S1B). Interestingly, there was a modest shift in the morphology of cells upon ALP treatment, whereby cells cultured with ALP appeared less spindle-shaped than the vehicle-treated controls (Supplementary Fig. S1C).

Having ruled out impaired cell survival, we posited that ALP-mediated changes in cytokine secretion may be a result of altered transcriptional programs. Thus, we utilized RT-qPCR to quantify RNA levels and found that ALP treatment led to marked reductions in *CCL2*, *CXCL12*, *IL6*, and *IL8* (Fig. 1C). We did not observe any significant changes in the RNA levels of *SERPINE1* in cells cultured with ALP (Supplementary Fig. S1D). We therefore elected to focus on the four most significantly altered cytokines (CCL2, CXCL12, IL6, and IL8) as a readout of ALP activity throughout the remainder of the study, which will be referred to collectively as the “ALP signature.”

Given that our previous study highlighted a structure-specific effect of BZDs on IL6 levels, we included five additional BZD compounds in our analyses. We found that midazolam was the only other BZD that phenocopied the ALP signature at both RNA and protein levels (Fig. 1C and D; Supplementary Fig. S1E).

We also evaluated the ALP signature in a murine CAF cell line (4175) as an orthogonal approach and observed significant reductions in *Ccl2*, *Cxcl12*, and *Il6* transcript levels following 24 hours of ALP treatment when compared with DMSO-treated control cells (Supplementary Fig. S1F). *IL8* was not paneled in this cell line as the murine genome lacks an *IL8* homolog. As proof of concept, we selected CCL2 and IL6 as representative targets to evaluate the effects of ALP on cytokine secretion and observed a consistent reduction in protein levels for both cytokines in the conditioned media of ALP-treated murine CAFs when compared with that of the vehicle control (Supplementary Fig. S1G–S1I). Interestingly, we also observed an ALP-dependent decrease in *CCL2*, *IL6*, and *IL8* RNA expression levels in human PDAC cells, suggesting that this signature may not be limited to CAFs (Supplementary Fig. S1J). Altogether, these data demonstrate that ALP suppresses immunomodulatory cytokine production *in vitro*.

### ALP abrogates IL6 production in murine TIF

To test this theory *in vivo*, we orthotopically injected syngeneic KPC1245 cells (derived from a female, murine Kras^LSL-G12D/+^; Trp53^LSL-R172H/+^; Pdx1-Cre pancreatic tumor) into the pancreata of 10-week-old female C57BL/6 mice, allowed 72 hours for postsurgical recovery, and began a 14-day intraperitoneal dosing regimen with 0.5 mg/kg ALP (*n* = 14) or DMSO equivalent (*n* = 13; Supplementary Fig. S2A). Consistent with our previous studies, ALP treatment was well tolerated but insufficient to reduce tumor burden relative to the vehicle-treated arm (Supplementary Fig. S2B and S2C). Although ALP treatment did not affect the tumor burden in chemo-naïve mice, we suspected that ALP could still induce changes in the production of proinflammatory cytokines known to influence response to therapy. We conducted ELISAs for murine CCL2 and IL6 using serum from whole blood as well as highly concentrated TIF. In the serum, we were unable to detect any notable changes in the concentration of either cytokine between the two experimental groups (Supplementary Fig. S2D and S2E). However, in the TIF, we observed that ALP-treated tumors generally exhibited lower concentrations of IL6 when compared with those from animals in the vehicle control arm (Supplementary Fig. S2F and S2G). These results support the notion that ALP dampens intratumoral cytokine production *in vivo*.

### Ligands of known ALP receptors only partially phenocopy ALP signature changes

**Figure 2.**
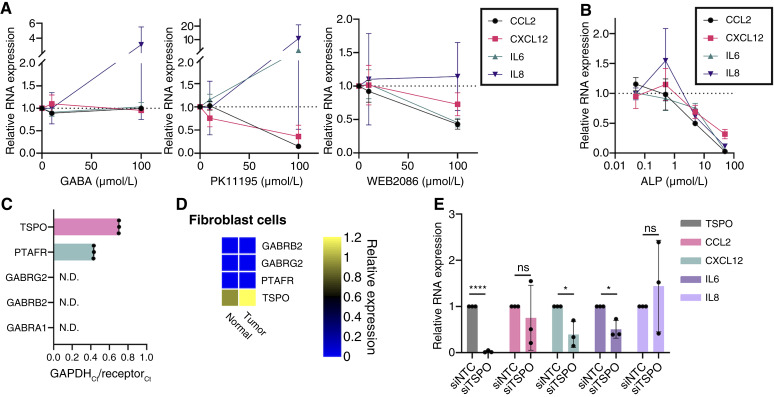
Ligands of known ALP receptors only partially phenocopy ALP signature changes. **A** and **B,** Fold change in RNA expression of respective cytokines as determined by RT-qPCR following 24 hours of treatment of C7-TA-PSC cells with **A**, 10 or 100 μmol/L of the indicated compound, or **B**, 0.05, 0.5, 5, or 50 μmol/L ALP relative to its vehicle control (0 μmol/L). **C,** Ratio of C_t_ values for each gene relative to that of *GAPDH* in C7-TA-PSC cells at baseline as determined by RT-qPCR. **D,** Heatmap of relative RNA levels in normal and tumor fibroblasts profiled in a human scRNA-seq dataset (26). **E,** Fold change in RNA expression for *TSPO* and ALP signature cytokines in siNTC and siTSPO transfected C7-TA-PSC cells as determined by RT-qPCR. Data in **A** were collected in technical duplicate and biological triplicate (*n* = 3). Data in **B**, **C**, and **E** were collected in technical and biological triplicate (*n* = 3). Data are represented as mean ± SD. N.D. indicates that C_t_ values for at least two technical replicates of each biological replicate were beyond the limit of detection. ns, not significant; *, *P* < 0.001; ****, *P* < 0.0001.

Although ALP is primarily considered a positive allosteric modulator of GABA-A receptor signaling, it has also been reported to have off-target effects on PTAFR and TSPO (36–39). In order to identify a predominant receptor through which ALP may be acting in the context of CAFs/inflammation, we designed an experiment to modulate all three of these receptors in the same manner in which ALP is reported to act on them. Briefly, we treated cells with vehicle, 10 or 100 μmol/L of a GABA-A agonist (GABA), TSPO ligand (PK11195), or PTAFR inhibitor (WEB2086) for 24 hours prior to collection for RT-qPCR analysis. Overall, GABA treatment led to negligible changes in cytokine transcript levels (Fig. 2A). However, treatment with both PK11195 and WEB2086 reduced RNA levels of *CCL2* and *CXCL12*. Although WEB2086 treatment also reduced *IL6* expression, PK11195 treatment led to a marked increase in the RNA levels of both *IL6* and *IL8*. It is important to note that neither 100 μmol/L PK11195 nor 100 μmol/L WEB2086 was sufficient to abrogate cytokine levels to the same degree as 50 μmol/L ALP (Fig. 2B).

We posited that some of these results may be attributable to the relative abundance of the target receptors in our model cell line. An RT-qPCR panel of *TSPO*, *PTAFR*, and the three most abundant GABA-A receptor subunits (α1β2γ2; refs. 17, 40, 41) revealed expression of *TSPO* and *PTAFR*, whereas GABA-A subunit RNA levels decreased below the limit of detection (Fig. 2C). In a publicly available human PDAC scRNA-seq dataset (26), there was low expression of the β2 and γ2 GABA-A subunits, with no detection of the α1 subunit among fibroblasts in both normal and tumor tissues (Fig. 2D). We also observed that *TSPO* expression far exceeded that of *PTAFR* and was elevated in the tumor fibroblasts relative to their normal counterparts in this dataset. With this in mind, we sought to further probe TSPO in our human CAFs using an siRNA-mediated knockdown system. siTSPO cells exhibited lower expression of *CXCL12* and *IL6* when compared with cells transfected with a siNTC (Fig. 2E). However, given that neither *TSPO* knockdown, PK11195, nor WEB2086 was sufficient to fully phenocopy ALP treatment, these data suggest that ALP may not be acting directly through TSPO or PTAFR in this context.

### Unbiased phosphoproteomics reveals ALP-dependent changes in kinase activity

**Figure 3.**
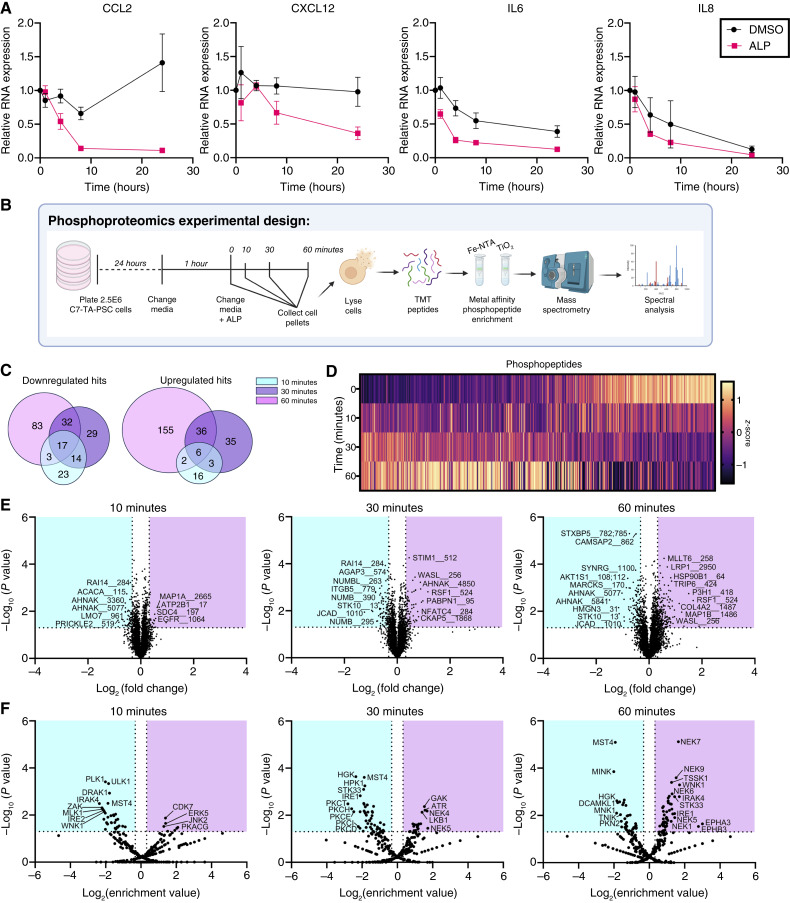
Unbiased phosphoproteomics reveals ALP-dependent changes in kinase activity. **A,** Fold change in cytokine RNA expression levels determined by RT-qPCR following treatment of C7-TA-PSC cells with 20 μmol/L ALP or DMSO for the indicated durations relative to the 0-hour vehicle control. Samples were analyzed in technical duplicate and biological triplicate (*n* = 3). **B,** Graphical representation of the phosphoproteomics experimental design, conducted in biological triplicate (*n* = 3). **C,** Venn diagrams representing the number of significantly upregulated or downregulated phosphopeptides upon 20 μmol/L ALP treatment at each designated time point relative to the 0-minute control. **D,** Heatmap depicting *z*-scores of changing phosphopeptides over time. **E,** Volcano plots of differentially enriched phosphopeptides upregulated (magenta) and downregulated (cyan) following 10, 30, and 60 minutes of 20 μmol/L ALP treatment relative to the 0-minute time point. **F,** Volcano plots of kinases in which activity is predicted to be enriched (magenta) or diminished (cyan) based on the differentially expressed phosphopeptide substrates at each time point.

In order to gain further insight into downstream pathways affected by ALP treatment in CAFs, we conducted time course analyses to identify how quickly changes in ALP signature cytokines could be detected. Refreshing the media at the time of treatment was sufficient to decrease IL expression in DMSO-treated cells within 4 hours, suggesting that the media composition alone affects cytokine transcription. However, most importantly, within 1 to 4 hours of 20 μmol/L ALP treatment, *CCL2*, *IL6*, and *IL8* RNA levels were markedly lower than those of the DMSO-treated cells, indicating that ALP directly induced rapid transcriptional reprogramming (Fig. 3A). With this in mind, we chose to conduct an unbiased phosphoproteomics screen with 0-, 10-, 30-, and 60-minute time points to identify ALP-dependent signaling cascades (Fig. 3B). Using mass spectrometry–based proteomics paired with immobilized metal affinity chromatography, we were able to detect 7,693 unique phosphosites (predominantly serines) on 6,541 phosphopeptides (predominantly monopeptides) across 2,433 phosphoproteins with a low coefficient of variance at each time point (Supplementary Fig. S3A–S3D). Overall, there was a time-dependent increase in the number of phosphorylation events (Fig. 3C–E). At the 10-minute time point, the most significant hits were downregulated when compared with the 0-minute baseline, whereas at 60 minutes, the majority of significant hits were upregulated phosphopeptides. Bulk GO analysis revealed enrichments in phosphoproteins involved in endocytosis, adherens junctions, and regulation of the actin cytoskeleton across all time points (Supplementary Fig. S3E). Although this finding is consistent with the morphologic changes in Supplementary Fig. S1C, it did not provide a direct explanation for the ALP-mediated cytokine changes. We next utilized kinase prediction software to infer common upstream regulators of our hits and observed an upregulation in inflammasome-related kinases at the 60-minute time point, a downregulation of protein kinase C family members at 30 minutes, and, most interestingly, a downregulation of kinases involved in TLR signaling at 10 minutes (Fig. 3F).

### ALP suppresses TLR4 signaling in PDAC CAFs

**Figure 4.**
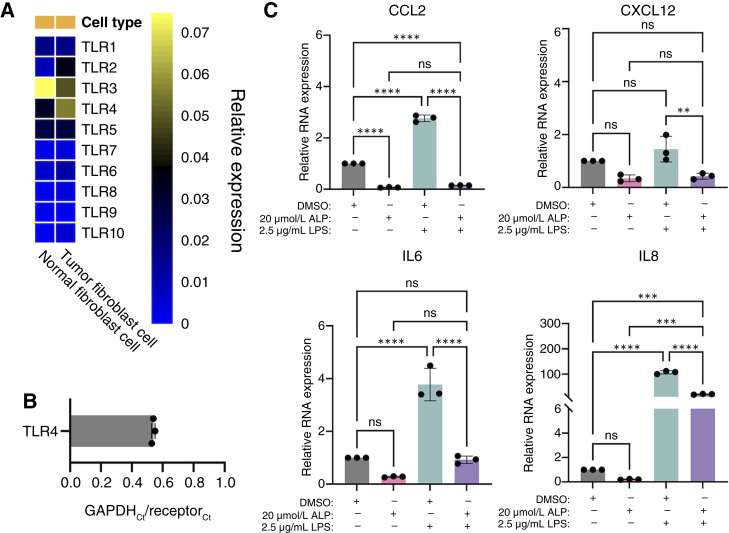
ALP suppresses TLR4 signaling in PDAC CAFs. **A,** Heatmap of relative RNA expression levels for TLRs in a scRNA-seq profile of human fibroblasts from normal pancreas and PDAC tumor tissues analyzed in ref. (26). **B,** RT-qPCR validation of *TLR4* RNA expression in C7-TA-PSC cells at baseline relative to *GAPDH*. **C,** Fold change in cytokine RNA expression levels determined by RT-qPCR following 24 hours of treatment with DMSO, 20 μmol/L ALP, and/or 2.5 μg/mL LPS. Experiments in **B** and **C** were conducted in technical and biological triplicate (*n* = 3). Data are represented as mean ± SD. Statistical analyses in **C** were conducted using one-way ANOVA with a Tukey correction for multiple comparisons. ns, not significant; **, *P* < 0.01; ***, *P* < 0.001; ****, *P* < 0.0001.

TLR signaling has a well-characterized role in the regulation of inflammation and, more specifically, inflammatory cytokine production (42, 43). The predicted downregulation of TLR-associated kinase activity following 10 minutes of ALP treatment led us to explore the landscape of these receptors in PDAC CAFs (44–48). Of the 10 known human TLR family members (44), we found that *TLR4* is the most highly expressed TLR among PDAC CAFs and is also enriched in tumor fibroblasts when compared with their normal counterparts (Fig. 4A; ref. 26). Although, much like the receptors investigated in Fig. 3, *TLR4* seems to be expressed in multiple cell subtypes within the PDAC TME, fibroblasts are among the highest expressors (Supplementary Fig. S4). We were also able to confirm *TLR4* expression in our human CAF cell line (Fig. 4B). As a result, we postulated that ALP may abrogate inflammatory cytokine production by dampening TLR4 activation in PDAC CAFs. In order to test this, C7-TA-PSC cells were treated with either DMSO or 20 μmol/L ALP, with or without 2.5 μg/mL LPS, a known TLR4 agonist. Although LPS treatment increased transcript levels of ALP signature genes relative to the vehicle condition, the combination of ALP with LPS significantly reduced these transcripts when compared with LPS alone (Fig. 4C). Altogether, these data suggest that ALP treatment is sufficient to dampen TLR4-mediated stimulation of inflammatory cytokine production in PDAC CAFs.

## Discussion

A defining feature of PDAC tumors is their dense, desmoplastic stroma that constitutes the vast majority of the tumor volume (49). This fibrotic tissue not only physically restricts immune cell infiltration but also contains diverse fibroblast subtypes that secrete intercellular signaling molecules contributing to immunosuppression (25, 50). In this study, we have demonstrated that the commonly prescribed BZD ALP elicits a potent reduction in inflammatory cytokines produced by PDAC CAFs *in vitro*, such as CCL2, CXCL12, IL6, and IL8.

We have found that the ALP signature identified in Fig. 1 is phenocopied by midazolam, but not all BZDs. This finding suggests that the structural similarities between these two compounds may bestow them with anti-inflammatory properties in this context. Although ALP is classified as a triazolo-BZD, midazolam is a fluoro-imidazo-BZD, meaning that both compounds contain an azole ring bound to the central BZD structure. Although diazepam and temazepam also contain N-substitutions, these compounds did not have a pronounced effect on cytokine expression, further supporting the notion that this phenotype is unique to only N-substituted azole-containing BZDs.

One explanation for the enhanced effects of ALP and midazolam could be their high binding affinity for the α1β2γ2 GABA-A receptor (18), the most abundant heteropentameric configuration of this receptor in the brain (40, 41). However, RT-qPCR of our model cell line, as well as mining of a human PDAC scRNA-seq dataset (26), revealed negligible expression of these GABA-A subunit genes in CAFs (Fig. 2C and D). These analyses do not exclude the possibility that these subunits are expressed at the protein level. It is also possible that CAFs express a less common combination of GABA-A subunits that are bound by ALP and/or midazolam. However, because GABA spike-in was insufficient to abrogate cytokine levels (Fig. 2B), it is less likely that ALP acts predominantly through GABA-A in PDAC CAFs.

In the 1980s, it was first reported that BZDs (ALP) can disrupt PTAFR signaling (36, 37). Although structurally distinct from BZDs, the azole-containing diazepine etizolam has been reported to demonstrate anti-inflammatory effects in esophageal CAFs via PTAFR (51). PTAFR has also recently been identified as a target for immunomodulation in PDAC (52). With this in mind, we tested a well-characterized PTAFR inhibitor (WEB2086) to see if this compound could recapitulate the ALP signature cytokine phenotype. Although 100 μmol/L WEB2086 was sufficient to decrease *CCL2*, *CXCL12*, and *IL6* transcript levels, we did not observe a reduction in *IL8* RNA (Fig. 2B). Although it is possible that ALP inhibits PTAFR to regulate some, but not all, of these cytokines, the low expression of *PTAFR* RNA in PDAC CAFs *in vitro* and *in vivo* suggests that this is less plausible. As with GABA-A expression, it is also possible that PTAFR is expressed at the protein level; however, further investigation is required in order to characterize the spatial distribution of this protein in PDAC tumors.

Another known off-target BZD binding partner is the TSPO (38, 39), also known as the peripheral BZD receptor. Although the binding affinity between ALP and TSPO is contested in the literature (53), biochemical analyses suggest that ALP does indeed bind TSPO with a K_i_ of ∼14 μmol/L (38). One recent report has linked TSPO to the regulation of inflammatory cytokine production in PDAC (54). Importantly, this study suggests that midazolam decreases plasma levels of CCL2 and IL6 in tumor-bearing mice and that this phenotype can be rescued with the TSPO ligand PK11195. Whether PK11195 acts predominantly as a TSPO agonist or antagonist is also contested in the literature (55). Because it has been reported that PK11195 mimics the effects of azole-containing BZDs (midazolam) on inflammation *in vitro* (56), we utilized this compound in our study to evaluate whether it could phenocopy the ALP results. Interestingly, although 100 μmol/L PK11195 recapitulated the decrease in *CCL2* and *CXCL12* that we observed upon ALP treatment, there was a marked increase in *IL6* and *IL8*. These results could potentially be explained by off-target effects of high-dose PK11195. Because genetic targeting of TSPO was also insufficient to reduce RNA expression of all ALP signature genes, it is more likely that there is a multifaceted receptor response to ALP.

In a PTAFR/TSPO agnostic manner, we conducted an unbiased mass spectrometry analysis that revealed time-dependent changes in protein phosphorylation downstream of TLR signaling. These results were of particular interest given the well-characterized role of TLRs in the regulation of intratumoral inflammation and immune signaling (57). Our analyses of scRNA-seq data suggested that TLR4 may be of particular interest in PDAC CAFs. Not only were TLR4 levels readily detectable in an immortalized PDAC CAF cell line, but they were also upregulated in tumor fibroblasts relative to normal pancreatic fibroblasts. TLR4 is a transmembrane protein that recognizes pathogen-associated molecular patterns from numerous endogenous and exogenous ligands (58). Importantly, although we utilized LPS as a potent TLR4 agonist, there are a number of proteins and molecules that are abundant in the PDAC TME with known TLR4 activity. It is possible that these factors may stimulate baseline cytokine production *in vitro* that is abrogated upon ALP treatment. It is also possible that off-target effects of ALP on PTAFR and TSPO may co-opt TLR4 signaling, as these pathways have also been implicated in TLR4-mediated inflammation (59–62). Ultimately, further studies are required to determine the predominant mechanism by which ALP interferes with TLR4 stimulus in PDAC CAFs.

It is important to note that the *in vitro* data presented herein should be interpreted in the context of the culture conditions. It is widely accepted that the substrate (i.e., plastic, Matrigel) chosen for CAF cultures can influence fibroblast phenotypes (63) and that these fibroblast phenotypes conversely influence substrate stiffness by remodeling the extracellular matrix (64). By culturing CAFs in a 2D monolayer, it is likely that our cells represent only a myofibroblast-like subpopulation of CAFs, which may not accurately represent the heterogeneity and plasticity of CAFs comprising the PDAC TME (65–67). As a result, we are actively investigating the effects of ALP on CAF phenotypes *in vivo*, including changes in extracellular matrix composition.

The data in the present study focus on the characterization of the effects of ALP on CAF cytokine output and provide a compelling basis to suggest that ALP may have a broad impact on remodeling the PDAC immune landscape and therapeutic response. For example, CCL2 is a key mediator of inflammatory monocyte recruitment in the PDAC TME, and targeting the CCL2/CCR2 axis has been shown to increase sensitivity to radio-, chemo-, and immunotherapies (68–71). Similarly, CXCL12, IL6, and IL8 contribute to complex stromal–immune paracrine signals in the TME (72) and are attractive therapeutic targets in PDAC (23, 73–77). Taken together, it is plausible that improved outcomes associated with ALP (17) may be attributable to the multifaceted anti-inflammatory CAF signaling program described herein.

## Supplementary Material

Supplementary Data Supplementary Data

Supplementary Figure S1 Supplementary Figure S1

Supplementary Figure S2 Supplementary Figure S2

Supplementary Figure S3 Supplementary Figure S3

Supplementary Figure S4 Supplementary Figure S4

Supplementary Table S1 Supplementary Table S1. Compound requisition and reconstitution information

Supplementary Table S2 Supplementary Table S2. Primer requisition information

## Data Availability

All data are contained within the article. Peptide analyses, kinase enrichment results, and all statistical analyses are included within the Supplementary Data files. Gene expression analyses from human PDAC scRNA-seq were obtained from datasets described in ref. (26). The mass spectrometry proteomics data have been deposited in the ProteomeXchange Consortium via the PRIDE partner repository (78) with the dataset identifier PXD067046 and 10.6019/PXD067046.
