# Supplementary material for: Alprazolam Reduces Inflammatory Cytokine Production in Pancreatic Cancer–Associated Fibroblasts
Source: Cancer Res Commun. 2026 May 6;6(5):1048–60. doi: 10.1158/2767-9764.CRC-25-0472 (PMC13147339; doi:10.1158/2767-9764.CRC-25-0472)
Supplement: Supplementary Figure S1 [file crc-25-0472_supplementary_figure_s1_suppsf1.pdf]

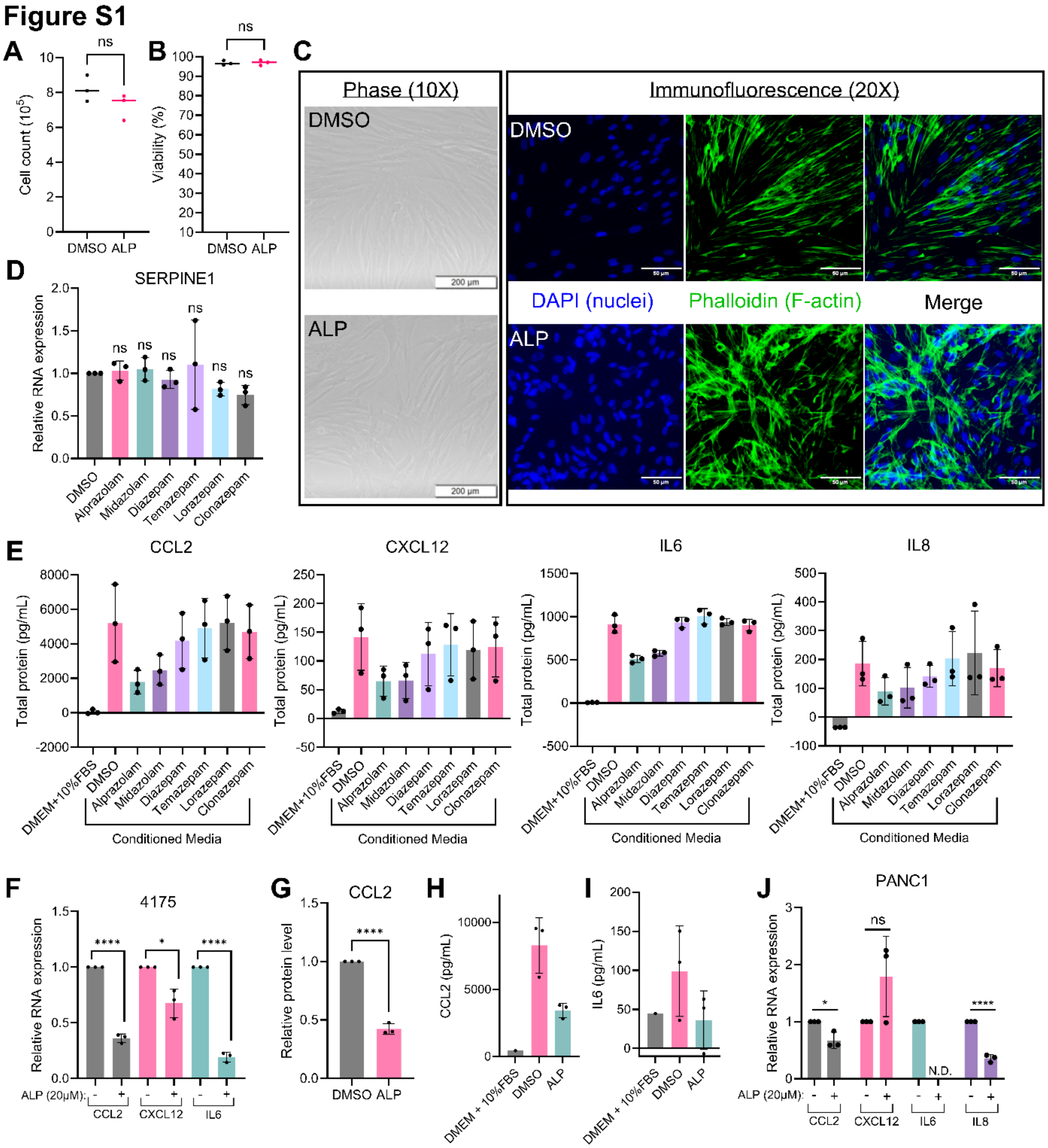

Supplementary Figure S1 **A**, absolute cell counts **B**, percent viability and **C**, representative phase contrast microscopy images at 10X magnification (left) or fluorescent phalloidin (green) and DAPI (blue) staining at 20X magnification (right) for C7-TA-PSC cells treated with 20μM ALP or DMSO for 24 hours. Datapoints in A-B correspond to average values of 50 reads for each biological replicate (n=3) with horizontal lines denoting the median. In C, Scale bars represent 200μm in 10X images and 50μm in 20X images. **D**, fold change in *SERPINE1* RNA expression as determined by RT-qPCR for cells treated as in A-C. **E**, absolute protein concentrations for cytokine levels detected by ELISA using FBS-rich media, or conditioned media from C7-TA-PSC cells treated with 20μM of each respective BZD or DMSO control for

24 hours. Data for D-E were collected in technical duplicate and biological triplicate (n=3). **F**, fold change in cytokine RNA expression in murine CAF cells (4175) treated with 20μM ALP or DMSO for 24 hours as determined by RT-qPCR. Data were collected in technical and biological triplicate (n=3). **G**, fold change in CCL2 protein levels and **H**, absolute CCL2 and **I**, IL6 concentrations detected by ELISA in FBS-rich media or conditioned media from 4175 cells as treated in F. Data for G-I were collected in technical duplicate and biological triplicate (n=3). **J**, fold change in cytokine RNA expression in PANC1 human PDAC cells treated with 20μM ALP or DMSO for 24 hours. Data were collected in technical and biological triplicate (n=3). N.D. indicates that C_t values for at least two technical replicates of each biological replicate were beyond the limit of detection. All data are plotted as mean ± standard deviation. Comparisons of two groups (A-B,F-G,J) were conducted using a two-tailed Student's t-test. Comparisons of more than two groups (D) were conducted using one-way ANOVA with a Dunnett's correction for multiple comparisons. *p < 0.05, **p < 0.01, ***p < 0.001, ****p < 0.0001.
