# Supplementary material for: Alprazolam Reduces Inflammatory Cytokine Production in Pancreatic Cancer–Associated Fibroblasts
Source: Cancer Res Commun. 2026 May 6;6(5):1048–60. doi: 10.1158/2767-9764.CRC-25-0472 (PMC13147339; doi:10.1158/2767-9764.CRC-25-0472)
Supplement: Supplementary Figure S2 [file crc-25-0472_supplementary_figure_s2_suppsf2.pdf]

Figure S2

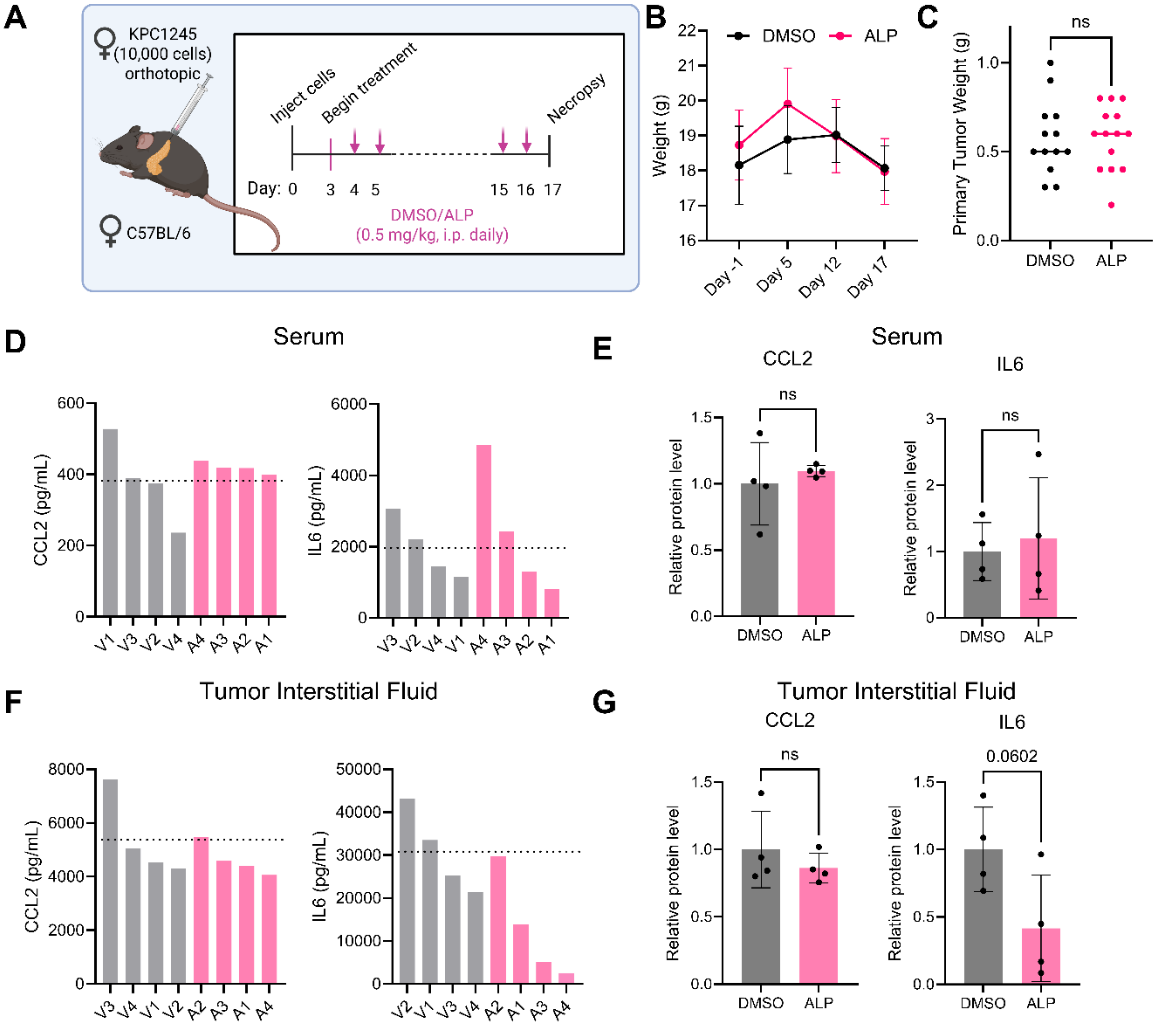

Supplementary Figure S2 **A**, experimental design for short-term intraperitoneal ALP treatment in a sex-matched, syngeneic, orthotopic PDAC mouse model. **B**, graph of mean body weight of vehicle (DMSO) and ALP-treated mice over time (DMSO: n=13, ALP: n=14). Data are plotted as mean ± standard deviation. **C**, dot plot depicting individual primary tumor weights at necropsy for mice in B. Horizontal lines depict median values. **D-G**, mean total (D,F) and relative (E,G) protein levels (from 2 technical replicates) of CCL2 (left) and IL6 (right) within the serum (D-E) and TIF (F-G) from 4 mice in each experimental arm (DMSO = “V” (grey), ALP = “A” (magenta)) as detected by ELISA. Each bar in D,F represents the mean total protein concentration for one individual mouse/tumor. Dotted lines in D,F represent the mean of the DMSO arm. Relative protein levels in E,G were normalized to the average of the DMSO group. Statistical analyses in C,E,G were conducted using a two-tailed Student’s t-test.
