# Supplementary material for: Alprazolam Reduces Inflammatory Cytokine Production in Pancreatic Cancer–Associated Fibroblasts
Source: Cancer Res Commun. 2026 May 6;6(5):1048–60. doi: 10.1158/2767-9764.CRC-25-0472 (PMC13147339; doi:10.1158/2767-9764.CRC-25-0472)
Supplement: Supplementary Figure S3 [file crc-25-0472_supplementary_figure_s3_suppsf3.pdf]

Figure S3

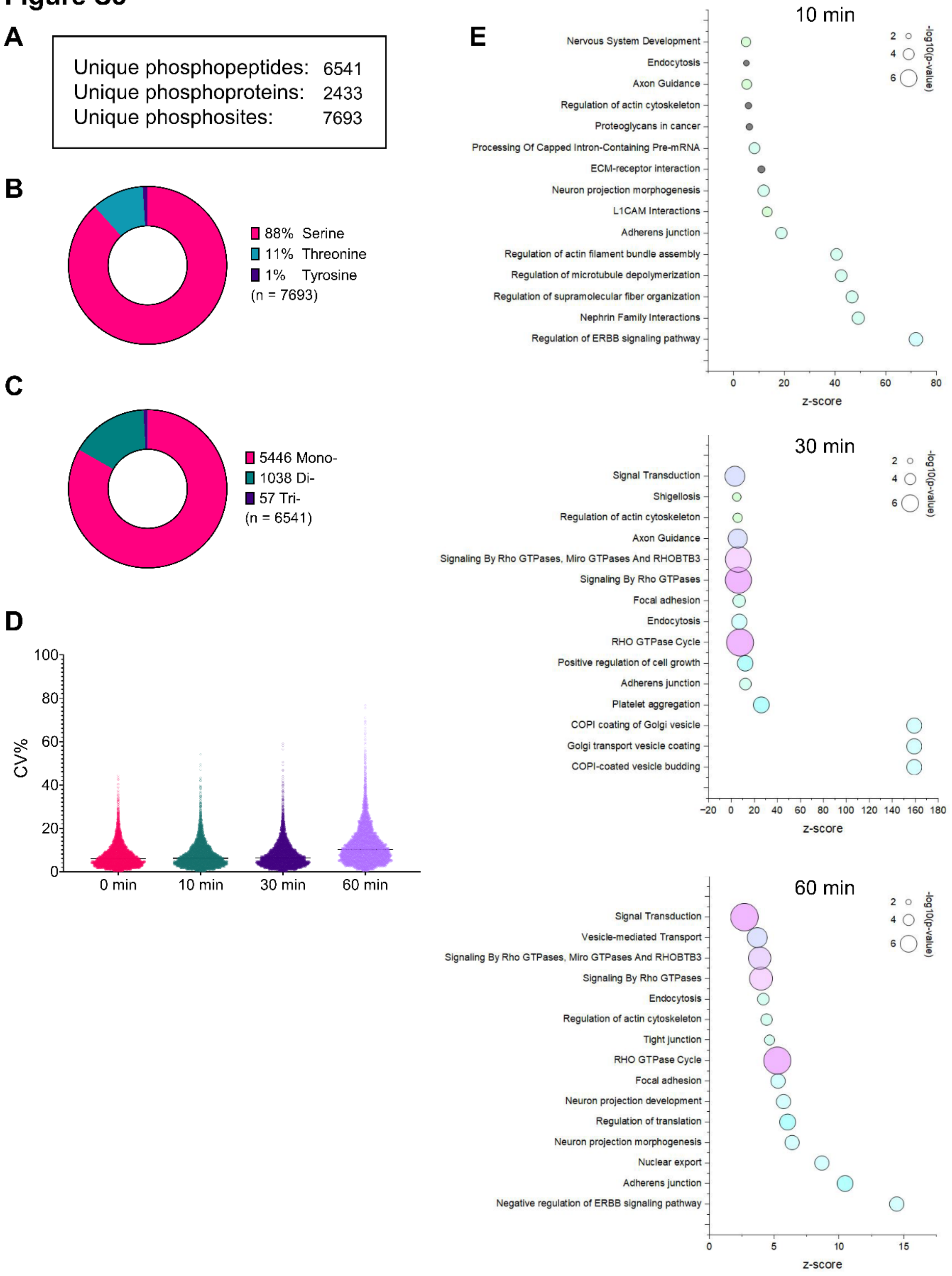

Supplementary Figure S3 **A**, description of unique phosphorylation events, **B**, phosphorylated amino acid representation, **C**, phosphorylated peptide distribution and **D**, coefficient of variation detected in the

phosphoproteomics screen. **E**, summary of top 15 most significant EnrichrKG gene ontology pathways (top 5 KEGG, GO, and Reactome) based on proteins with differentially expressed phosphopeptides at each timepoint after 20μM ALP treatment relative to the 0 minute control in human PDAC CAF cells (C7-TA-PSC).
