# Supplementary material for: Alprazolam Reduces Inflammatory Cytokine Production in Pancreatic Cancer–Associated Fibroblasts
Source: Cancer Res Commun. 2026 May 6;6(5):1048–60. doi: 10.1158/2767-9764.CRC-25-0472 (PMC13147339; doi:10.1158/2767-9764.CRC-25-0472)
Supplement: Supplementary Figure S4 [file crc-25-0472_supplementary_figure_s4_suppsf4.pdf]

Figure S4

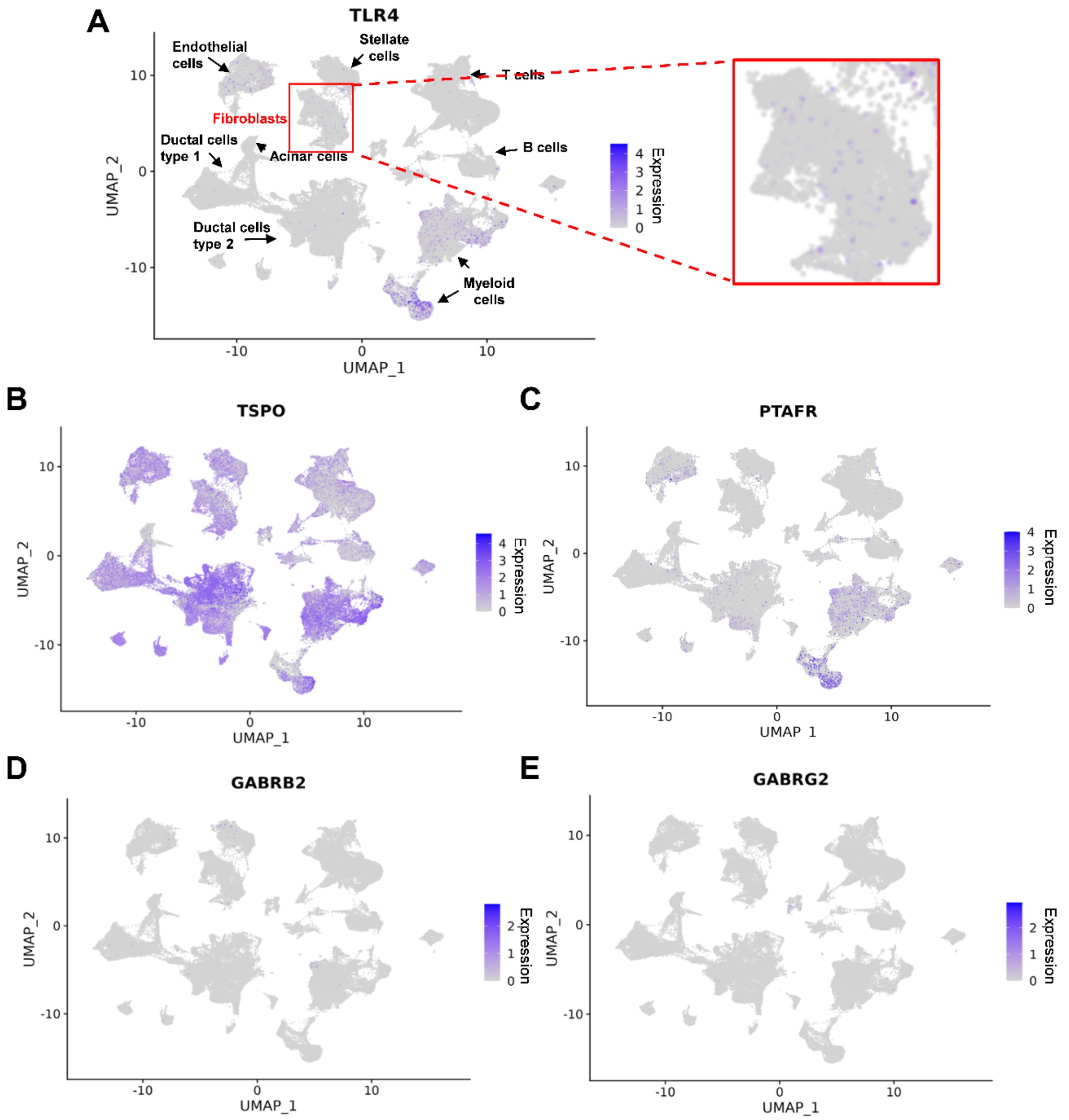

Supplementary Figure S4 UMAP plots of **A**, *TLR4*, **B,** *TSPO*, **C**, *PTAFR*, **D**, *GABRB2*, and **E**, *GABRG2* RNA expression in single cells from both normal and tumor-bearing pancreata reported in (26). Red box in A shows higher resolution view of *TLR4* expression in fibroblasts. Color density corresponds to expression of the indicated gene.
