# Supplementary material for: Alprazolam Reduces Inflammatory Cytokine Production in Pancreatic Cancer–Associated Fibroblasts
Source: Cancer Res Commun. 2026 May 6;6(5):1048–60. doi: 10.1158/2767-9764.CRC-25-0472 (PMC13147339; doi:10.1158/2767-9764.CRC-25-0472)
Supplement: Supplementary Table S1 — Compound requisition and reconstitution information [file crc-25-0472_supplementary_table_s1_suppst1.pdf]

Supplementary Table S1 Compound requisition and reconstitution information

Compound	Vendor	Catalog Number	Diluent	Stock Concentration	Storage Temperature (ºC)
Alprazolam	Cayman Chemical	14255	DMSO	10mM	-20
Midazolam	Cayman Chemical	16193	DMSO	10mM	-20
Diazepam	Cayman Chemical	15887	DMSO	10mM	-20
Temazepam	Cayman Chemical	15918	DMSO	10mM	-20
Lorazepam	Sigma-Aldrich	L1764	DMSO	10mM	-20
Clonazepam	Cayman Chemical	14263	DMSO	10mM	-20
GABA	Sigma-Aldrich	A2129	Water	100mM	-80
WEB2086	Cayman Chemical	14532	DMSO	10mM	-80
PK11195	Cayman Chemical	10525	DMSO	100mM	-80
LPS	Thermo-Fisher Scientific	00-4976-93	–	2.5mg/mL	-20
