# Supplementary material for: Alprazolam Reduces Inflammatory Cytokine Production in Pancreatic Cancer–Associated Fibroblasts
Source: Cancer Res Commun. 2026 May 6;6(5):1048–60. doi: 10.1158/2767-9764.CRC-25-0472 (PMC13147339; doi:10.1158/2767-9764.CRC-25-0472)
Supplement: Supplementary Table S2 — Primer requisition information [file crc-25-0472_supplementary_table_s2_suppst2.pdf]

Supplementary Table S2 Primer requisition information

**Gene Target**	**Species**	**Catalog Number**
GAPDH	*Homo sapiens*	qHsaCED0038674
CCL2	*Homo sapiens*	qHsaCID0011608
CXCL12	*Homo sapiens*	qHsaCID0012398
IL6	*Homo sapiens*	qHsaCED0044677
IL8	*Homo sapiens*	qHsaCED0023767
SERPINE1	*Homo sapiens*	qHsaCID0006432
GAPDH	*Mus musculus*	qMmuCED0027497
CCL2	*Mus musculus*	qMmuCED0048300
CXCL12	*Mus musculus*	qMmuCID0019961
IL6	*Mus musculus*	qMmuCED0045760
TSPO	*Homo sapiens*	qHsaCED0042769
PTAFR	*Homo sapiens*	qHsaCED0004563
GABRA1	*Homo sapiens*	qHsaCID0006684
GABRB2	*Homo sapiens*	qHsaCID0008972
GABRG2	*Homo sapiens*	qHsaCID0008338
TLR4	*Homo sapiens*	qHsaCED0037607
